# Does the EyeChart App for iPhones Give Comparable Measurements to Traditional Visual Acuity Charts?

**DOI:** 10.22599/bioj.146

**Published:** 2020-04-15

**Authors:** Katie Ansell, Gail Maconachie, Anne Bjerre

**Affiliations:** 1 The University of Sheffield, GB

**Keywords:** Visual acuity, Smartphone technology, EyeChart app

## Abstract

**Aim:**

To investigate if the EyeChart app gives accurate visual acuity (VA) measurements that are comparable to those achieved using traditional VA charts.

**Method:**

Twenty-four participants (aged 18–27 years, mean 20.13 ± 1.78 years) with VA of 6/60 Snellen or better regardless of any strabismus, amblyopia, or ocular pathology volunteered for this prospective study. The best-corrected monocular VA of each participant’s right eye was measured on the Snellen chart at 6 m, the ETDRS chart at 3 m, and the EyeChart app presented on an iPhone SE at 1.2 m (4ft).

**Results:**

The mean VA scores obtained were: –0.13 ± 0.08 logMAR on the Snellen chart, –0.11 ± 0.08 logMAR on the ETDRS chart, and –0.09 ± 0.07 logMAR on the EyeChart app. After Bonferroni Correction adjustments were applied, a significant difference was found between the EyeChart app and the Snellen chart (t = –3.756, p = 0.003), however the difference between the EyeChart app and the ETDRS chart did not reach statistical significance (t = –2.391, p = 0.076). The EyeChart app had a strong correlation with both the Snellen (r = 0.79, p < 0.01) and ETDRS charts (r = 0.88, p < 0.01). The Coefficients of Agreement revealed a variation of less than one logMAR line between the EyeChart app and the traditional VA charts (Snellen: 0.09 logMAR; ETDRS: 0.08 logMAR).

**Conclusion:**

This study found that the EyeChart app gives accurate VA scores that are comparable to those achieved using the gold-standard ETDRS chart in a healthy young adult population. However, the accuracy and repeatability of the EyeChart app when testing a patient population must be investigated before it can be integrated into clinical practice.

## Introduction

Testing visual acuity (VA) is one of the most sensitive ways of monitoring the functioning of the visual system (Levenson & Kozarsky 1990), therefore it is vital to ensure any chart used to measure VA is accurate and repeatable. The Snellen chart, first developed in 1862, is still commonly used for testing VA in clinical practice (Kaiser 2009) despite lacking an accurate scoring system (McGraw, Winn & Whitaker 1995). To provide more standardisation and accuracy in the scoring process the logMAR Bailey-Lovie chart was developed using a family of 10 non-serif letters (Bailey & Lovie 1976). The Early Treatment Diabetic Retinopathy Study used the same logMAR chart design principles but a family of 10 Sloan letters to develop the ETDRS chart (Kassoff 1979) which is now considered the gold-standard chart for VA testing (Kalpana, Karthick & Jayarajini 2013).

Some research has already been published into the accuracy of VA apps for smartphones with mixed results. Bastawrous et al. (2015) found the Peek Acuity app had a strong correlation with both the ETDRS and Snellen charts, with greater similarities between the app and the ETDRS chart. Yu et al. (2014) tested smartphone-based electronic visual acuity (SEVA) technology on patients with a range of VAs and ocular pathologies and found it highly correlated to the ETDRS chart and near LEA numbers test with no significant difference between the VA scores achieved. Perera et al. (2015) found that the Snellen iPhone app gave no significant difference in mean VA scores compared to the Snellen chart, although the largest difference of nearly three lines was recorded when VA was worse than 6/18 Snellen.

Differing outcomes have also been reported when using iPads as an alternative presentation method for VA testing. Zhang et al. (2013) found that with VAs better than 6/60 Snellen the EyeChart pro iPad app compared well with the Snellen chart but with VAs poorer than 6/60 Snellen the app gave significantly worse results of nearly one line. Alternatively, Gounder et al. (2014) reported that the EyeSnellen iPad app had very good agreement with the Snellen chart, and Black et al. (2013) highlighted that, provided glare is minimised, iPads can produce VA measurements that are ‘indistinguishable’ from gold-standard charts.

Many other healthcare services have begun to experience significant benefits from using technology (Mosa, Yoo & Sheets 2012; Zvornicanin, Zvornicanin & Hadziefendic 2014) including the standardisation and expansion of services. The introduction of smartphone apps for VA testing could therefore have a profound impact on the Ophthalmic professions by providing an opportunity to improve the standard of care provided to patients outside the traditional clinical setting.

Technology offers a promising way to standardise the services provided by those without extensive Ophthalmic training as both the Peek Acuity app (Bastawrous et al. 2015) and Paxos app (Pathipati et al. 2016) have been shown to give accurate VA measurements even when used by non-medically trained staff. Building on these results, if VA apps can be shown to be accurate when used by professionals with a range of training, they could lead to better inter-observer agreement within Ophthalmology departments and provide a greater continuity of care to patients.

The portability of this technology could also be exploited for use outside the clinical setting in order to expand the reach of Ophthalmic services into situations where traditional VA charts are typically not available. VA apps for smartphones could be used in homes and community clinics (Bastawrous et al. 2015) and the testing distance of four feet means they can also be used to more easily assess patients in hospital wards (Perera et al. 2015). If smartphone apps can be shown to give VA results that accurately compare to those achieved using the gold-standard VA chart, they can be employed to ensure patients who are unable to be assessed in the traditional setting are not disadvantaged and receive a comparative level of care to those seen in the main hospital clinic. Apps for VA testing could also be used alongside smartphone technology for indirect ophthalmoscopy and fundus photography (Haddock & Qian 2015) to provide accurate and efficient Ophthalmic screening in developing countries as some of the main risk factors for avoidable sight loss are poverty and poor access to healthcare (Cook et al. 2006).

The EyeChart app, unlike many other apps for VA testing, is free to download, making it appealing to a variety of groups including patients wanting to monitor their own VA between appointments, in addition to charities working in developing countries and managers of services within the NHS both keen to take advantage of the benefits of this technology without having to allocate limited funds that could be spent elsewhere. The easy accessibility of the EyeChart app means it has already been downloaded over 1 million times despite there being no published research into its accuracy. The aim of this study was therefore to investigate if the EyeChart app for iPhones gives accurate VA measurements that are comparable to those achieved using the traditional Snellen chart, on which its design is based, and the gold-standard ETDRS chart in a young adult population.

## Methodology

### Participants

The protocol for this experiment adhered to The Declaration of Helsinki and received approval from The University of Sheffield Ethics Committee. All volunteers gave informed written consent before participating in the study.

Twenty-four (one male, 23 female) undergraduate Orthoptic students aged 18–27 years (mean age 20.13 ± 1.78 years) from The University of Sheffield were recruited for this prospective study. This study only aimed to record the VA of each participant’s right eye, meaning those with strabismus, amblyopia, or any ocular pathology were eligible to participate provided the VA in their right eye was 6/60 Snellen or better at 6 m. Participants with refractive errors were required to wear their refractive correction throughout testing. Paediatric volunteers under 18 years of age were excluded from participating, as were any presbyopic individuals needing reading glasses.

### Equipment

The app used for this study was the EyeChart app for iPhones (available at: https://itunes.apple.com/us/app/eyechart-vision-screening/id293163439) which presents a Sloan letter chart in a Snellen format and gives VA scores in metric Snellen fractions. The EyeChart app was presented on an iPhone SE at 1.2 m (4 ft), the wall-mounted Snellen chart was presented at 6 m, and the ETDRS chart was presented at 3 m. All three VA charts complied with the British Standards for VA chart luminance (BSI 2003).

### Design and Procedure

The study was a repeated-measures design meaning each participant had the VA of their right eye tested on all three charts using a counterbalanced testing order. The Snellen chart used to ensure participants fitted the inclusion criteria was different to the chart used during the main experiment to avoid learning effects.

Standardised experimental testing conditions were used including black-out blinds in the testing room. The VA charts were hidden from view when not in use and then presented to the participants one at a time in a pre-determined randomised order. When using the EyeChart app, the phone screen was positioned away from sources of glare as this has been shown to affect the visibility of the chart therefore reducing the accuracy of the results obtained (Black et al. 2013). To minimise any fatigue effects, participants were given a 30 second rest period between testing on the different VA charts.

All participants were told to read the smallest line of letters they could see on each VA chart using standardised instructions and they also received the same level of interaction and encouragement throughout testing. The best-corrected monocular VA of the right eye was tested to threshold and recorded using a letter-by-letter scoring system to improve the accuracy of the results obtained (Bailey & Lovie-Kitchin 2013). The VA scores from the ETDRS chart were directly recorded in logMAR values. When using the EyeChart app and the Snellen chart, the VA scores were recorded in metric Snellen fractions and the number of letters seen on the threshold line was noted. Each letter was given a score based on the number of optotypes on that line, taking into account any lines missing from the charts used, and this allowed partial Snellen lines to be converted into equivalent letter-by-letter logMAR scores.

### Statistical Analysis

A critical p-value ≤ 0.05 was used to indicate statistical significance while a critical p-value ≤ 0.01 indicated the results were highly statistically significant.

A one-factor repeated-measures ANOVA and paired-scores t-tests were performed to determine if the mean VA scores achieved using each chart were significantly different. As multiple t-tests were performed, the Bonferroni Correction adjustment was applied to reduce the risk of a type one error occurring.

The correlation between the VA charts was identified using scatter plots and quantified using Pearson Correlation Coefficients. Bland-Altman plots were used to explore the agreement between the VA charts, and Coefficients of Agreement (1.96 times the standard deviation of the mean difference) were calculated to discover the expected clinical difference between them.

## Results

Of the 24 participants, two were hypermetropic (+1.50 to +4.00 DS), 13 were myopic (–0.75 to –4.75 DS), and nine were emmetropic. Nine of the participants also had astigmatism (–0.25 to –2.25 DC). None of the participants had any manifest strabismus, amblyopia, or ocular pathology.

The average VA scores achieved were: –0.13 ± 0.08 logMAR on the Snellen chart, –0.11 ± 0.08 logMAR on the ETDRS chart, and –0.09 ± 0.07 logMAR on the EyeChart app (Table 1). A one-factor repeated-measures ANOVA revealed a significant difference between at least two of the VA charts used (F_2,46_ = 8.220, p < 0.001). After applying the Bonferroni Correction adjustment, paired-scores t-tests revealed a statistically significant difference between the EyeChart app and the Snellen chart (t = –3.756, p = 0.003), however the difference between the EyeChart app and the ETDRS chart (t = –2.391, p = 0.076) and the difference between the Snellen chart and the ETDRS chart (t = –1.904, p = 0.208) did not reach statistical significance.

**Table 1:** The descriptive statistics performed on the data collected from each VA chart type.

	Snellen chart VA score (logMAR)	ETHRS chart VA score (logMAR)	EyeChart app VA score (logMAR)

Mean	–0.128	–0.110	–0.091
Standard Deviation	0.077	0.083	0.072
Standard Error	0.016	0.017	0.015
Range	0.214	0.260	0.225

**Figure 1:**
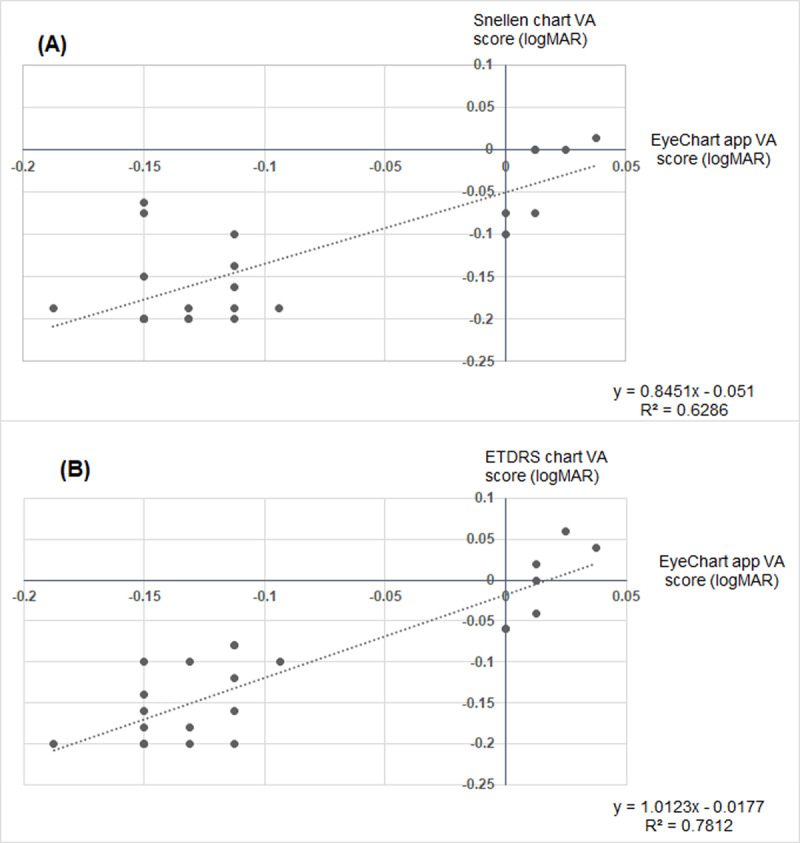
Scatter plots comparing: **(A)** the VA scores achieved on the EyeChart app (on the horizontal axis) to the VA scores achieved on the Snellen chart (on the vertical axis); and **(B)** comparing the VA scores achieved on the EyeChart app (on the horizontal axis) to the VA scores achieved on the ETDRS chart (on the vertical axis).

Scatter plots were produced to examine the relationship between the EyeChart app and the Snellen Chart (Figure 1a) and between the EyeChart app and the ETDRS chart (Figure 1b). Pearson Correlation Coefficients revealed that the EyeChart app had a strong positive correlation with both the Snellen chart (r = 0.79, p < 0.01) and the ETDRS chart (r = 0.88, p < 0.01). However, the negative intercepts on both scatter plots (Figure 1a: –0.05 logMAR; Figure 1b: –0.02 logMAR) suggest that on average the EyeChart app slightly underestimated VA compared to the traditional VA charts.

**Figure 2:**
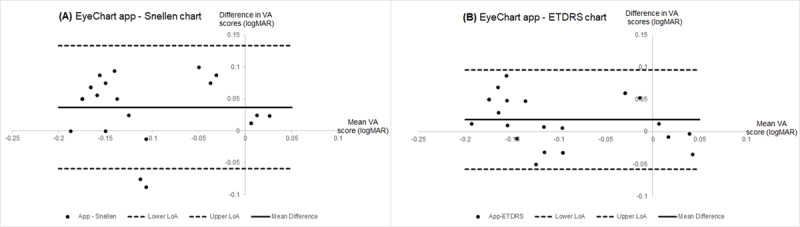
Bland-Altman plots to show: **(A)** the agreement between the VA scores achieved on the EyeChart app and the VA scores achieved on the Snellen chart; and **(B)** the agreement between the VA scores achieved on the EyeChart app and the VA scores achieved on the ETDRS chart. NB: Abbreviation used – LoA = limit of agreement.

The Bland-Altman plots show the level of agreement between the EyeChart app and the Snellen chart (Figure 2a) and between the EyeChart app and the ETDRS chart (Figure 2b). In Figure 2a the mean difference is positive (0.04 ± 0.05 logMAR) and all data points bar five lie above the horizontal axis indicating that for almost all participants the EyeChart app gave poorer VA scores than the Snellen Chart and for one participant this difference was as large as 0.10 logMAR (one logMAR line). In Figure 2b the mean difference is also positive (0.02 ± 0.04 logMAR) and, although data points lie above and below the horizontal axis, slightly more points lie above the axis indicating that on average the EyeChart app also gave poorer VA scores than the ETDRS chart. The Coefficient of Agreement between the EyeChart app and the Snellen Chart was 0.09 logMAR and between the EyeChart app and the ETDRS chart it was 0.08 logMAR. The 95% limits of agreement (LoA) between the EyeChart app and the ETDRS chart (–0.06 to 0.096 logMAR) are narrower than those between the EyeChart app and the Snellen chart (–0.06 to 0.13 logMAR).

## Discussion

This study found that the VA scores achieved using the EyeChart app are significantly different to those obtained using the Snellen chart but are comparable to those obtained using the gold-standard ETDRS chart in a healthy young adult population.

When comparing the EyeChart app to the Snellen chart, all statistical tests indicate the difference between the two charts is equivalent to approximately half a logMAR line (0.05 logMAR). Alternatively, when comparing the EyeChart app to the ETDRS chart, the statistical tests reveal a closer clinical comparison with a difference between the two charts equivalent to just one logMAR optotype (0.02 logMAR). This is supported by the Pearson Correlation Coefficients which indicate a stronger correlation between the EyeChart app and the ETDRS chart (r = 0.88) than between EyeChart app and the Snellen chart (r = 0.79).

Despite the ETDRS-type Peek Acuity app (Bastawrous et al. 2015) and the Snellen-type Paxos app (Pathipati et al. 2016) being comparable to both the Snellen and ETDRS charts, stronger agreements were seen between the apps and the traditional VA chart they were based on. This is in contrast to the results of this study, which found the Snellen-type EyeChart app to be comparable to the ETDRS chart whilst significantly differing from the Snellen chart. It is important to note that this study used a small sample size meaning the validity of the results obtained are limited by the amount of data collected. However, direct comparisons of the results of this study to those of previous studies are also limited due to the different experimental set-ups. For example, Bastawrous et al. (2015) investigated Tumbling E optotypes and Pathipati et al. (2016) presented single optotypes with patients indicating the correct answer using a matching card. Another key difference is the populations used in the studies. Pathipati et al. (2016) conducted their experiment on patients in an emergency eye department and Bastawrous et al. (2015) tested their app in homes and community clinics in rural Kenya, again limiting the comparisons that can be drawn from the results of the studies.

The Coefficients of Agreement calculated to compare the EyeChart app to the traditional VA charts were less than 0.10 logMAR when testing a normal adult population. As this study was only able to recruit ocular normals and the range of VAs tested was small, future studies should aim to include a wider range of VAs and those with ocular pathology because other apps for VA testing have been shown to give significantly different results in groups of patients with reduced VAs despite being comparable when testing those with satisfactory VA (Perera et al. 2015; Zhang et al. 2013). If a similar level of agreement can be replicated when using the EyeChart app to test the demographic of patients seen in clinic this would have significant implications. These findings would suggest that a difference of less than one logMAR line could be expected if the EyeChart app was used interchangeably with the Snellen and ETDRS charts in clinical practice. This is much narrower than the currently reported 0.15 logMAR general variability of VA measurements between clinics (Siderov & Tiu 2000) which is important when clinical decisions are based on VA measurements, for example when deciding if a patient is eligible for cataract surgery, or when deciding if a patient meets the legal standard of VA required for driving.

This study found that the EyeChart app can give accurate VA scores when participants are wearing their refractive correction. To ensure the closer testing distance does not adversely affect the results obtained when refractive errors are uncorrected, the EyeChart app must be shown to be equally sensitive at detecting reduced VA caused by both uncorrected myopia and hypermetropia.

Furthermore, before it could be introduced as a clinical VA assessment tool, it would be important to establish both the intra- and inter-examiner repeatability of the EyeChart app in addition to a value for the test-retest variability in both normal and patient populations as these will help examiners to know if any change in VA detected using the EyeChart app is true.

The Snellen chart is being used less in clinic in favour of logMAR and electronic alternatives (Bailey & Jackson 2016) because the standardised design of the ETDRS chart has caused it to supersede the Snellen chart and become the gold-standard VA chart for Ophthalmic professionals (Ferris & Bailey 1996). While the differences between the EyeChart app and the Snellen chart are important to note, any potential new VA chart must be compared to the gold-standard. Therefore, more weight should be given to the results comparing the EyeChart app to the ETDRS chart, and this study has found the agreement between the VA scores achieved on the two charts to be less than one logMAR line.

## Conclusion

This study found that the VA scores achieved on the EyeChart app are comparable to those achieved using the gold-standard ETDRS chart in a healthy young adult population. This is a promising finding; however, it is necessary for further large-scale studies to investigate the EyeChart app when used on a variety of patient population groups. Only with this additional data could the use of the EyeChart app in clinical practice be advocated and the possible opportunities this may present to improve the Ophthalmic services provided to patients could then be considered.
